# Supplementary material for: Identification of intracellular cavin target proteins reveals cavin-PP1alpha interactions regulate apoptosis
Source: Nat Commun. 2019 Jul 22;10:3279. doi: 10.1038/s41467-019-11111-1 (PMC6646387; doi:10.1038/s41467-019-11111-1)
Supplement: Supplementary file 1 — Supplementary Information [file 41467_2019_11111_MOESM1_ESM.pdf]

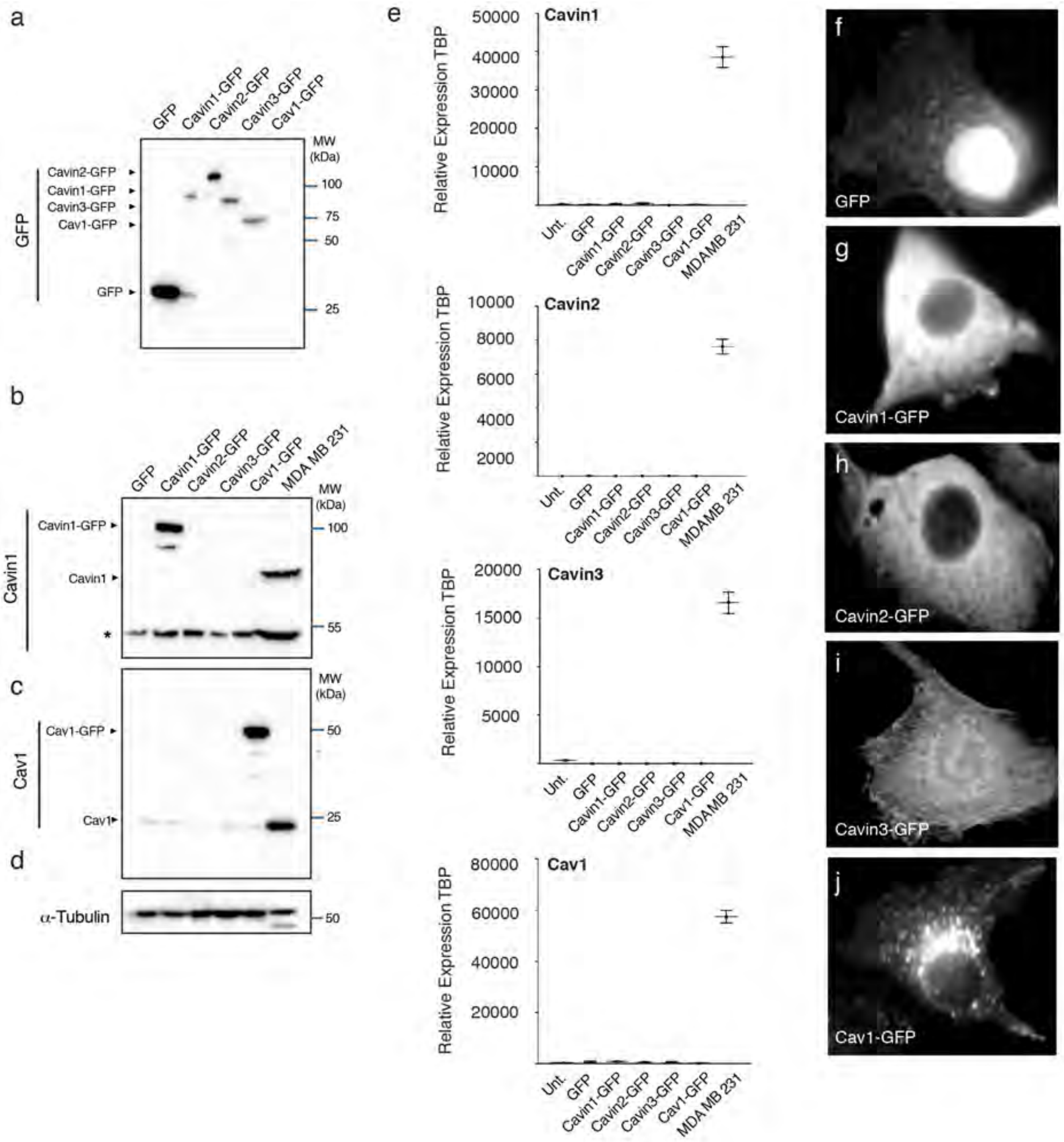

Supplementary Figure 1. Localization of the cavin proteins and CAV1 in MCF-7 cells.

(a). Western blot analysis of MCF-7/GFP, MCF-7/Cavin1-GFP, MCF-7/Cavin2-GFP, MCF-7/Cavin3-GFP, MCF-7/CAV1-GFP and MDA-MB231 cells as a positive control for (a) GFP, (b) Cavin1, (c) CAV1 and (d) Tubulin as the loading control. * denotes a non-specific band.

(e). Total mRNA expression of the human endogenous Cavin1, Cavin2, Cavin3 or CAV1 proteins as determined by RT-PCR in the Methods section in Untransfected (Unt.), GFP, Cavin1-GFP, Cavin2-GFP, Cavin3-GFP, CAV1-GFP stably expressing MCF-7 cells and in MDA-MB231 cells as a control presented as mean ± SD for three independent experiments.

(f-j). Representative images of the localization of GFP alone (f) as a control and each of the cavin proteins, Cavin1-GFP (g), Cavin2-GFP (h), Cavin3-GFP (i) and CAV1-GFP (j) are shown from three independent experiments.

a Ingenuity pathway analysis

Top Pathways	*p* value	Ratio	
Glycolysis	9.18E-06	4/41	0.073
Gluconeogenesis	7.77E-04	2/48	0.048

Mol and Cell Functions	*p* value	
Cell Death and Survival	2.22E-07 - 4.44E-02	22 mols
Celluar Compromise	5.70E-07 - 4.40E-02	11 mols
Protein Synthesis	4.68E-06 - 3.69E-02	13 mols

b ALPHAScreen Cavin1-interacting proteins

c ALPHAScreen Cavin3-interacting proteins

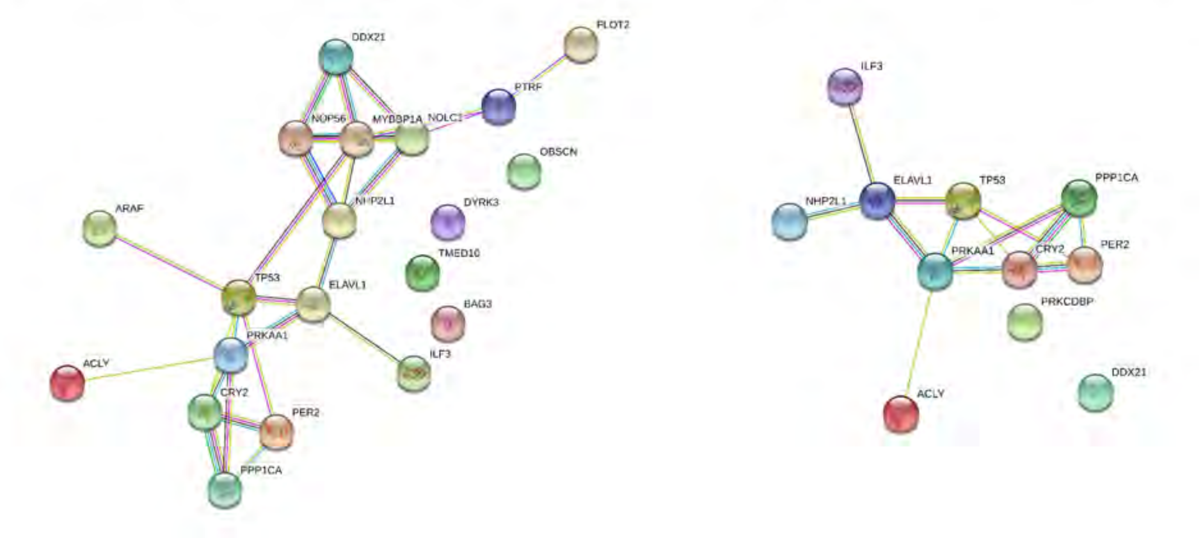

Supplementary Figure 2. Pathway analysis for Cavin3 potential interacting proteins.

(a). All potential Cavin3 interacting proteins identified by BioID-mass spectrometry (Source data are provided as a Source Data file) were analyzed by Ingenuity Pathway Analysis. The Top pathways and Molecular (Mol) and Cellular (Cell) functions for Cavin3 were identified including relevant *p* values and molecule numbers (mols) as indicated.

(b-c). STRING diagrams were generated of the identified interacting proteins from the ALPHAScreen for (b) Cavin1 and (c) Cavin3.

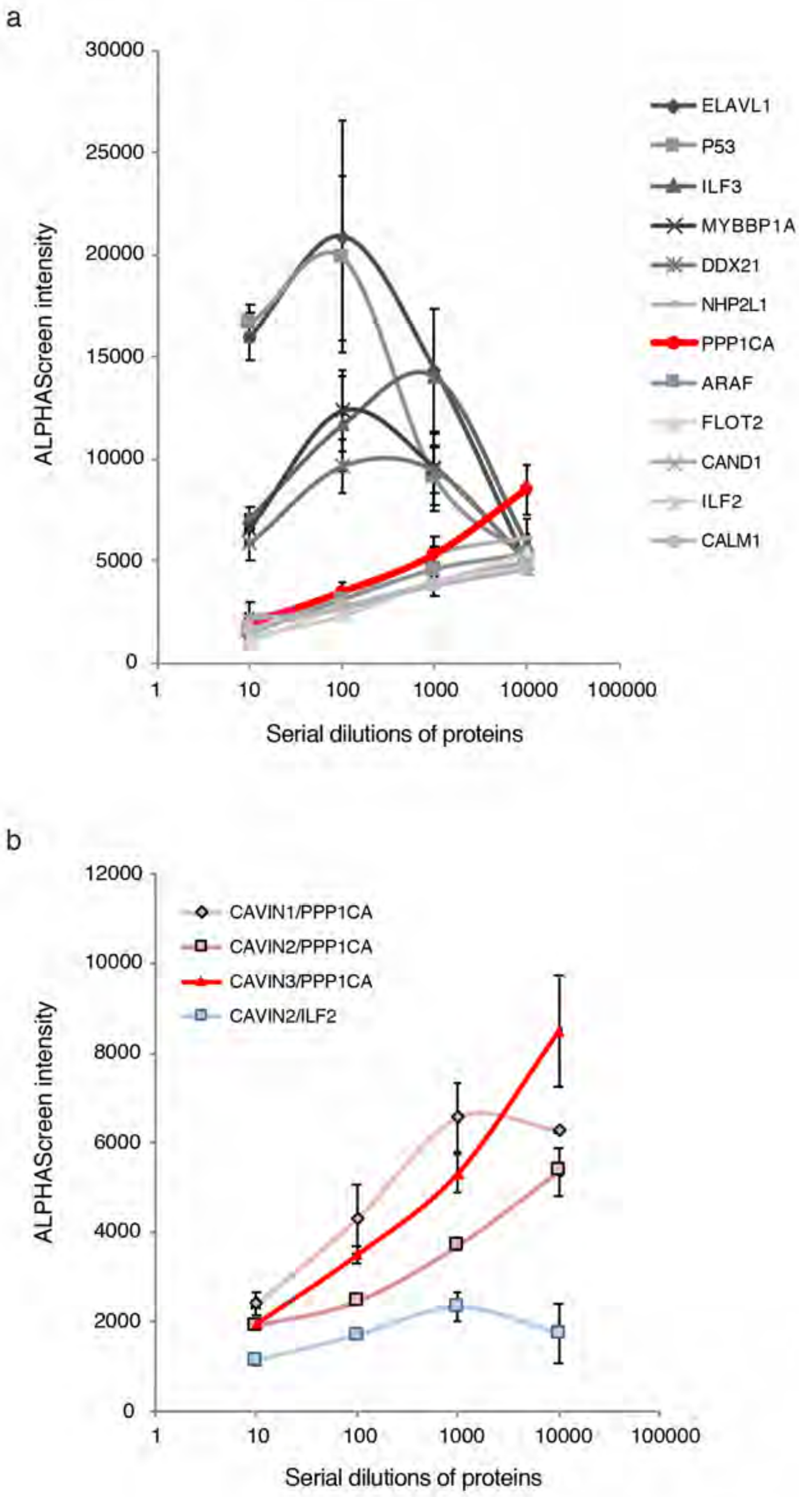

Supplementary Figure 3. ALPHAScreen intensity response for cavin interacting proteins.

(a). ALPHAScreen intensity response of serial dilutions of Cavin3 (1-100000) with a selection of tested proteins, ELAV1, P53, ILF3, MYBBP1A, DDX21, NHP2L1, PPP1CA (red), ARAF, FLOT2, CAND1 and ILF2.

(b). ALPHAScreen intensity response of serial dilutions of each of the cavin proteins, Cavin1/PPP1CA, Cavin2/PPP1CA and Cavin3/PPP1CA and Cavin2/ILF2 (control). Data presented in (a) and (b) were calculated from three biological replicates with three technical replicates for each sample.

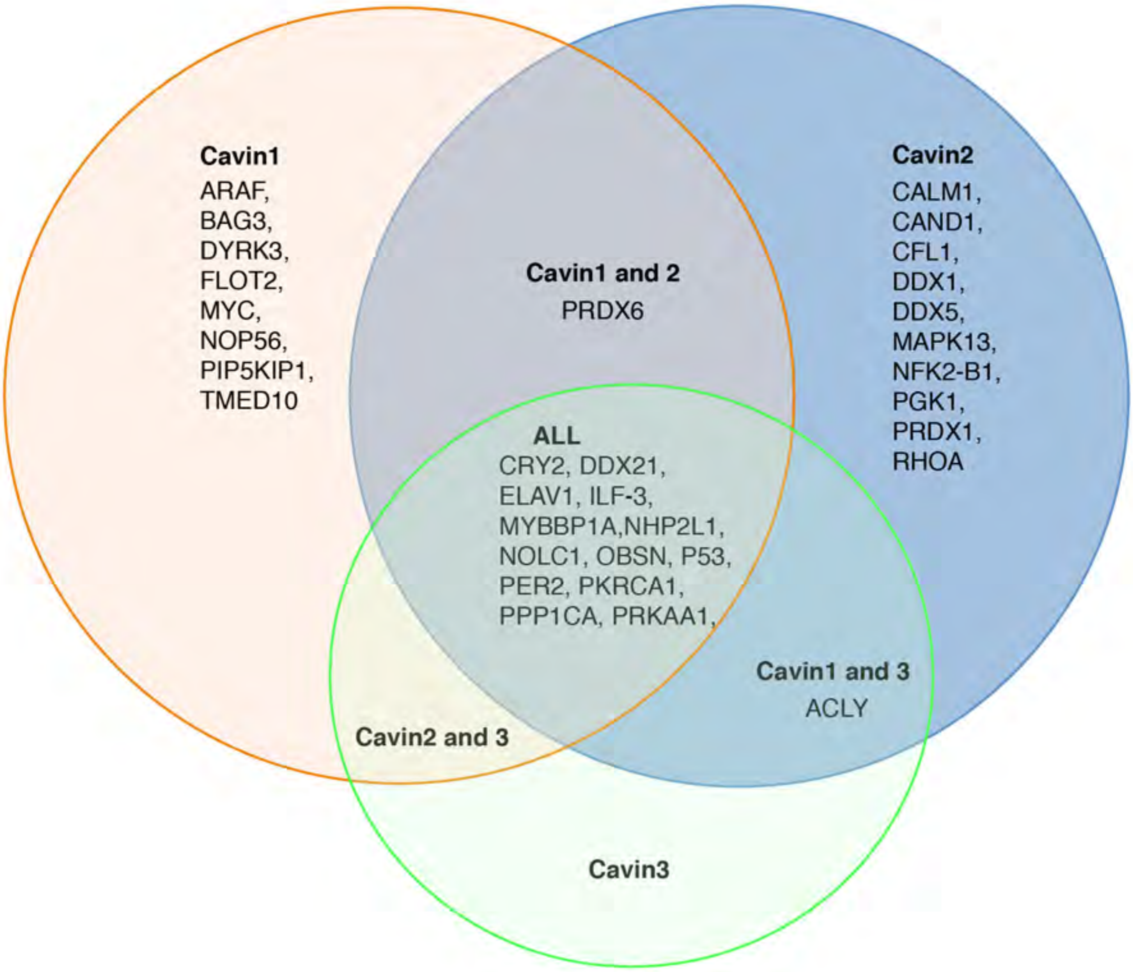

Supplementary Figure 4. Potential non-caveolar cavin interacting proteins.

Proportionate Venn diagram where blue represents Cavin1 only interacting proteins, orange represents Cavin2 only interacting proteins, green represents Cavin3 only interacting proteins with ALL representing proteins that showed a positive interaction with each of Cavin1, Cavin2 and Cavin3 independently. (Source data are provided as a Source Data file)

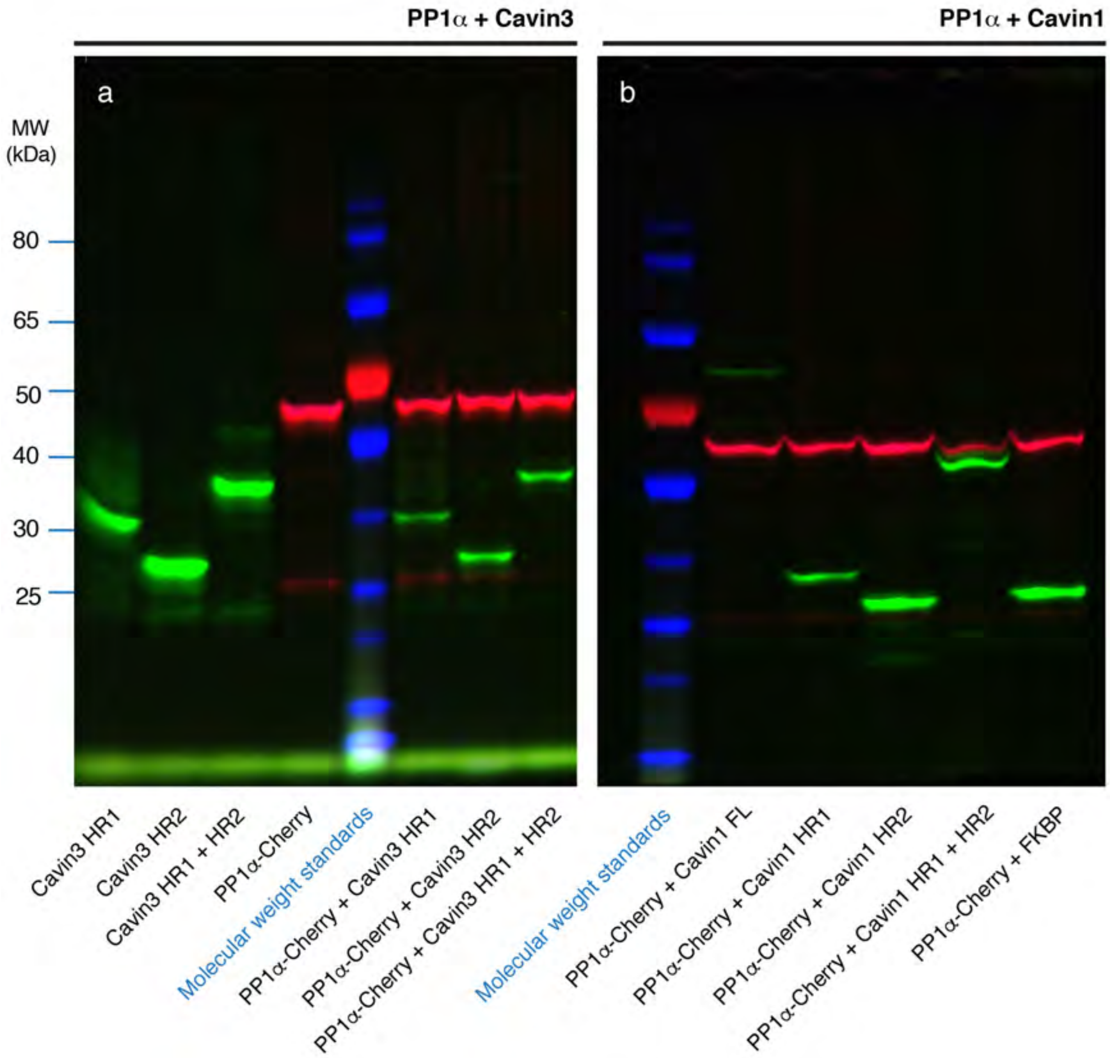

Supplementary Figure 5. ***Leishmania*** **Translation of Cavin3-PP1α and Cavin1-PP1α.**

(a). Representative in-gel fluorescence SDS-PAGE analysis of *Leishmania* translated Cavin3 HR1-GFP alone, Cavin3 HR2-GFP alone, Cavin3 HR1+HR2-GFP alone and PP1α-Cherry alone, molecular weight standards, and co-expression of PP1α-Cherry and Cavin3 HR1-GFP, PP1α-Cherry and Cavin3 HR2-GFP and PP1α-Cherry and Cavin3 HR1+HR2-GFP.

(b). Representative in gel fluorescence SDS-PAGE analysis of *Leishmania* coexpression of PP1α-Cherry and Cavin1 Full length (FL)-GFP, PP1α–Cherry and Cavin1 HR1-GFP, PP1α–Cherry and Cavin1 HR2-GFP, PP1α–Cherry and Cavin1 HR1+HR2-GFP and PP1α–Cherry and Cavin1 FKBP as a control.

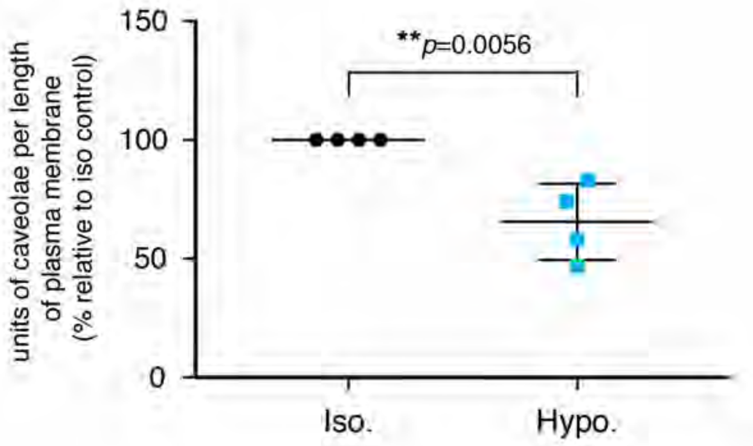

Supplementary Figure 6. Quantitation of caveolar number following hypo-osmotic treatment.

A431 cells under isotonic and hypo-osmotic conditions were fixed and imaged for electron microscopy. The number of caveolae per length of plasma membrane were then quantified. % relative to isotonic control group (isotonic control 100 ± 0% versus hypo-osmotic treatment 65.5± 16.1%) are presented for four sets of experiments where **p= 0.0056 using Student t-test (two tailed).

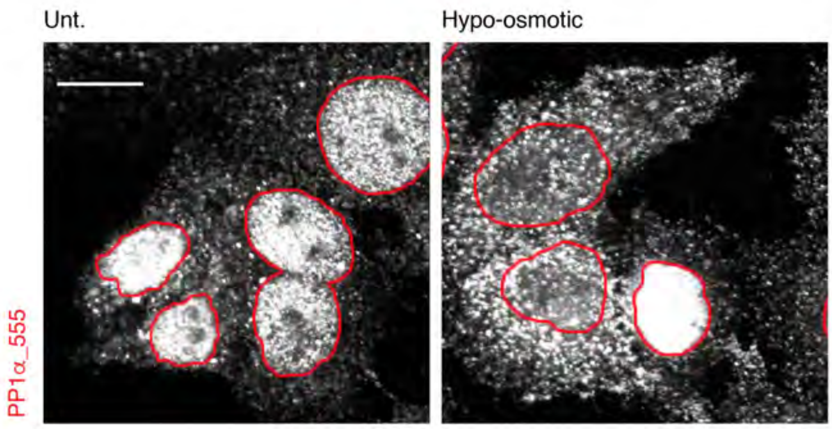

Supplementary Figure 7. PP1 translocation to the cytosol following hypo-osmotic treatment in MCF-7 cells.

MCF7 cells treated with hypo-osmotic medium were fixed for immunofluorescence of PP1α visualised under confocal microscopy. Images were inverted to grey scale and the nuclei were outlined in red. Scale bar, 10 μm.

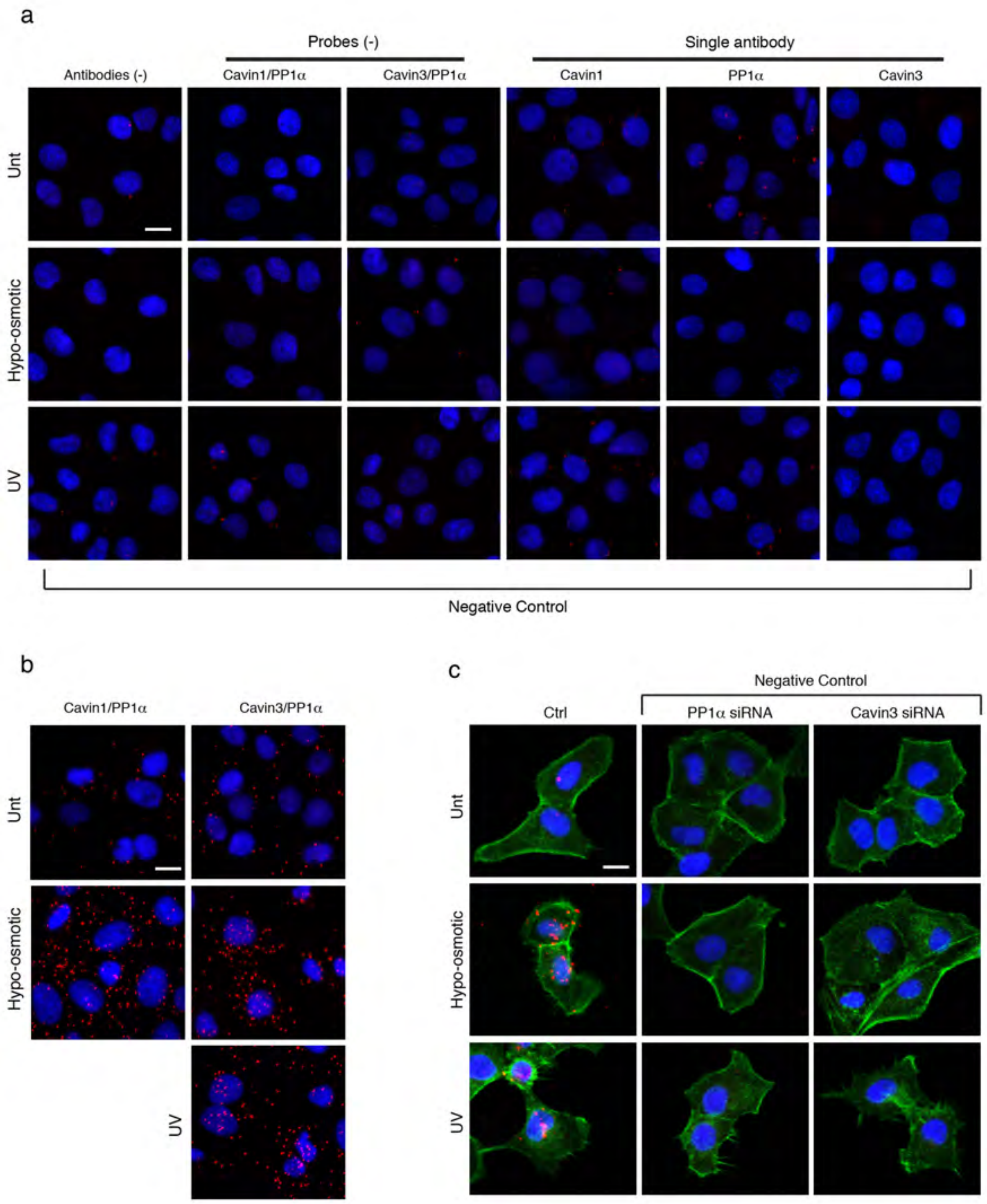

Supplementary Figure 8. PLA controls.

(a-b). Fluorescence microscopy analysis of PLA signals generated in A431 cells either left untreated or treated with hypo-osmotic medium or UV treatment in the absence of primary antibodies, PLA probe controls or individual Cavin1, Cavin3 and PP1α antibody as negative controls for PLA signals with the existence of probes and Cavin3/PP1α or Cavin1/PP1α antibody pairs. Representative images are from at least two independent experiments as shown.

(c). PLA controls following knockdown of PP1α and Cavin3. Three independent experiments were performed. Scale bar, 10 μm.

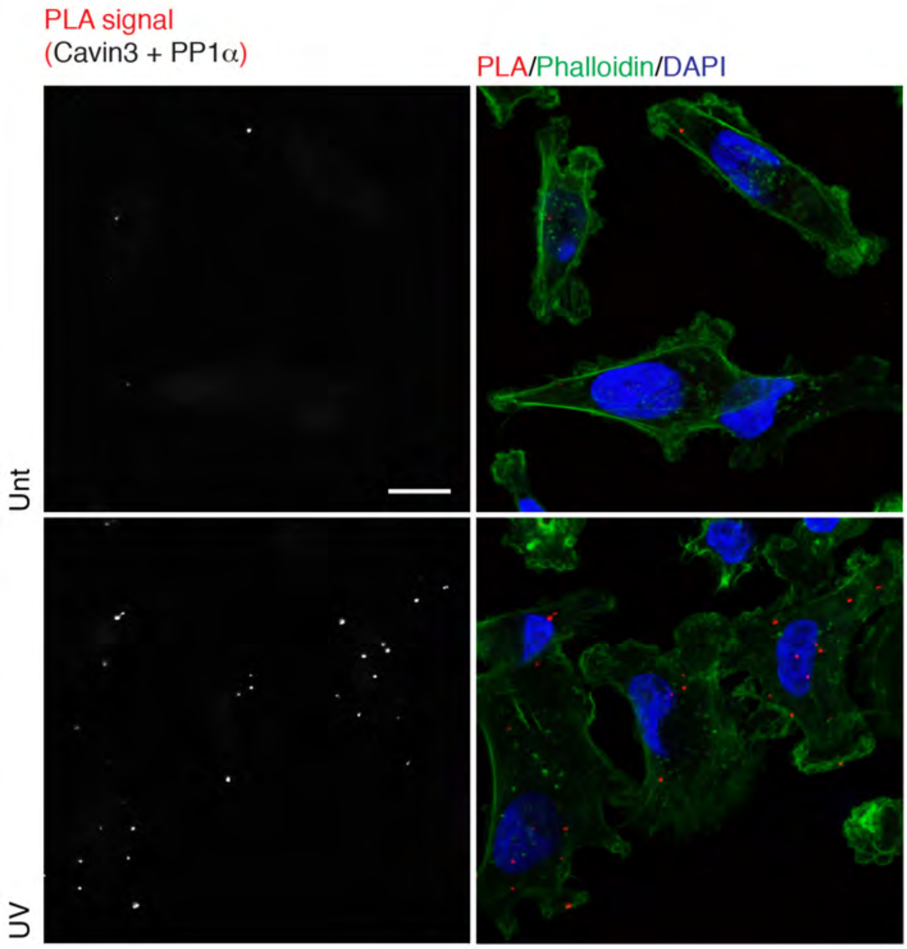

Supplementary Figure 9. Association of Cavin3 and PP1α with UV treatment in MDA-MB231 cells.

Confocal microscopy images represent the PLA signals in MDA-MB231 cells with and without UV treatment. PLA signals alone is presented as inverted images (left panel). The merged images include PLA signal (red), Alexa Fluor 488-Phalloidin (green) and DAPI (blue) channels. Phalloidin staining was used to identify the borders of cells. The nucleus is indicated by DAPI staining. Scale bar, 10 μm. Images are representative of two independent experiments performed.

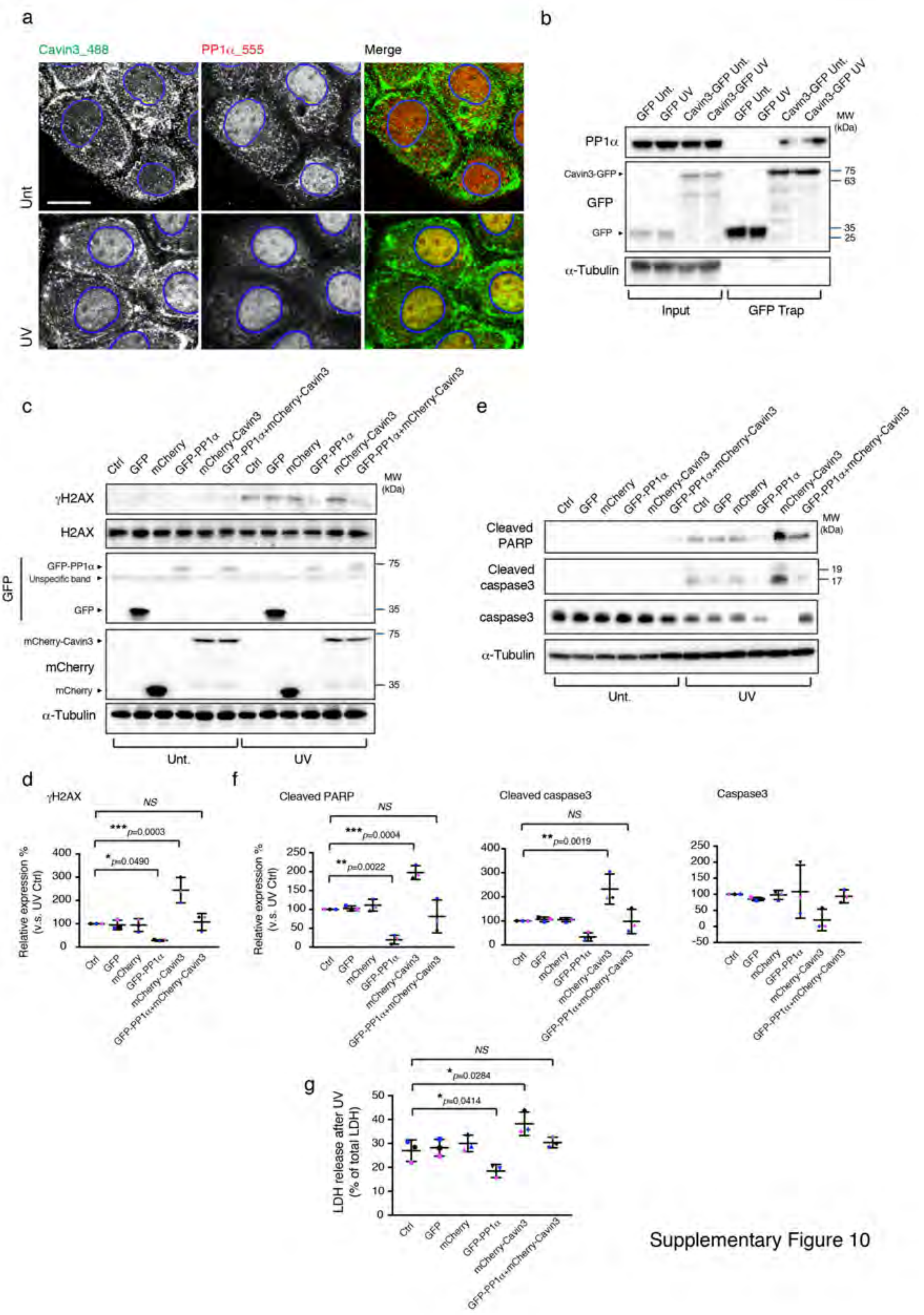

Supplementary Figure 10. PP1α and Cavin3 possess opposite roles in UV-induced apoptosis.

(a). Representative immunofluorescence images of Cavin3 (green) and PP1α (red) in untreated or UV treated A431 cells from three independent experiments. DNA was outlined by blue circle. Scale bar, 10 µm.

(b). GFP Trap assays of A431 cells transfected with GFP-Cavin3 or GFP with and without UV treatment western blotted with anti-PP1α antibodies. Transfection efficiency was confirmed with GFP antibodies. α-Tubulin was used as the loading control. Western blots are representative of three independent experiments.

(c). Western blots with anti-γH2AX antibodies of A431 cells untransfected/control (Ctrl), transfected with GFP-vector (GFP) or mCherry-vector (mCherry), GFP-PP1α, mCherry-Cavin3 or GFP-PP1α + mCherry-Cavin3. Transfection efficiency was confirmed with GFP and Cherry antibodies. α-Tubulin was used as the loading control.

(d). Relative protein expression (%) of γH2AX in untransfected/Ctrl and transfected A431 cells with GFP, mCherry, GFP-PP1α, mCherry-Cavin3 or GFP-PP1α + mCherry-Cavin3 from three independent experiments (Ctrl vs GFP-PP1: *p=0.0490, Ctrl vs mCherry-Cavin3: ***p=0.0003; one-way ANOVA, Tukey's multiple comparison test).

(e). Western blots of cleaved PARP, cleaved caspase 3 and total caspase 3 in A431 cells left untransfected (Ctrl), transfected with GFP or mCherry, GFP-PP1α, mCherry-Cavin3 or GFP-PP1α + mCherry-Cavin3. α-Tubulin were used as the loading control.

(f). Relative protein expression (%) of cleaved PARP (Ctrl vs GFP-PP1: **p=0.0022, Ctrl vs mCherry-Cavin3: ***p=0.0004), cleaved caspase 3 (Ctrl vs mCherry-Cavin3: **p=0.0019) and caspase 3 in untransfected (Ctrl), transfected with GFP, mCherry, GFP-PP1α, mCherry-Cavin3 or GFP-PP1α+mCherry-Cavin3 from three independent experiments (one-way ANOVA using Tukey's multiple comparison test).

(g). LDH release as a % of the total LDH following UV treatment was calculated where Ctrl vs GFP-PP1: *p=0.0414, Ctrl vs mCherry-Cavin3: *p=0.026. Data is represented as mean ± SD of triplicate experiments. *p*-value (one-way ANOVA) versus UV control cells.

Figure 2a

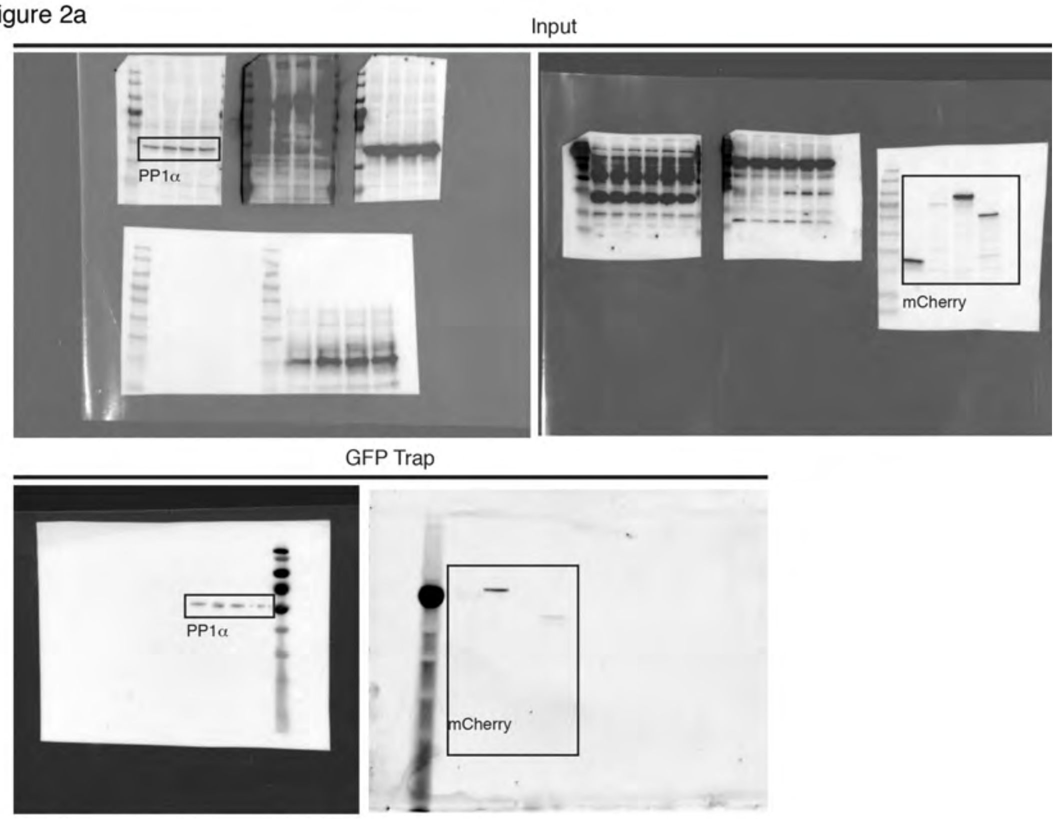

Figure 2b

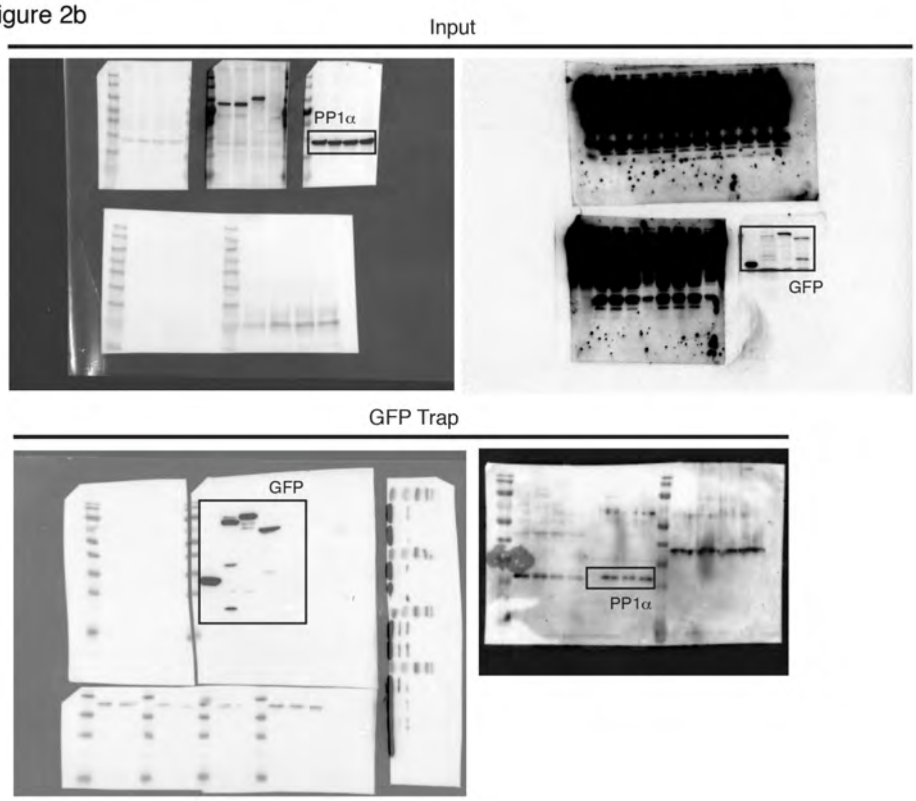

Supplementary Figure 11. Uncropped and unprocessed scans of the western blots.

Figure 3d

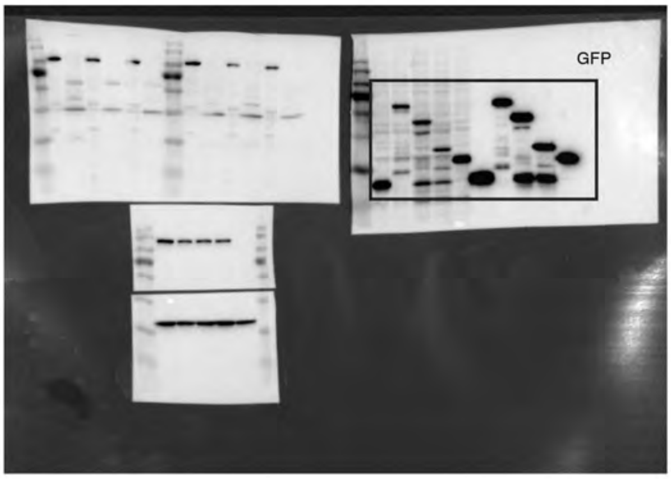

Figure 3e

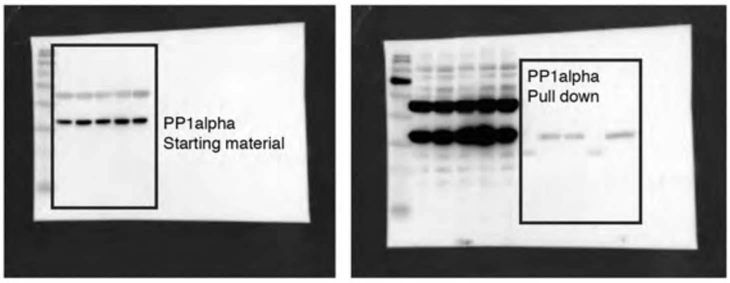

Supplementary Figure 11. Uncropped and unprocessed scans of the western blots.

Figure 5d

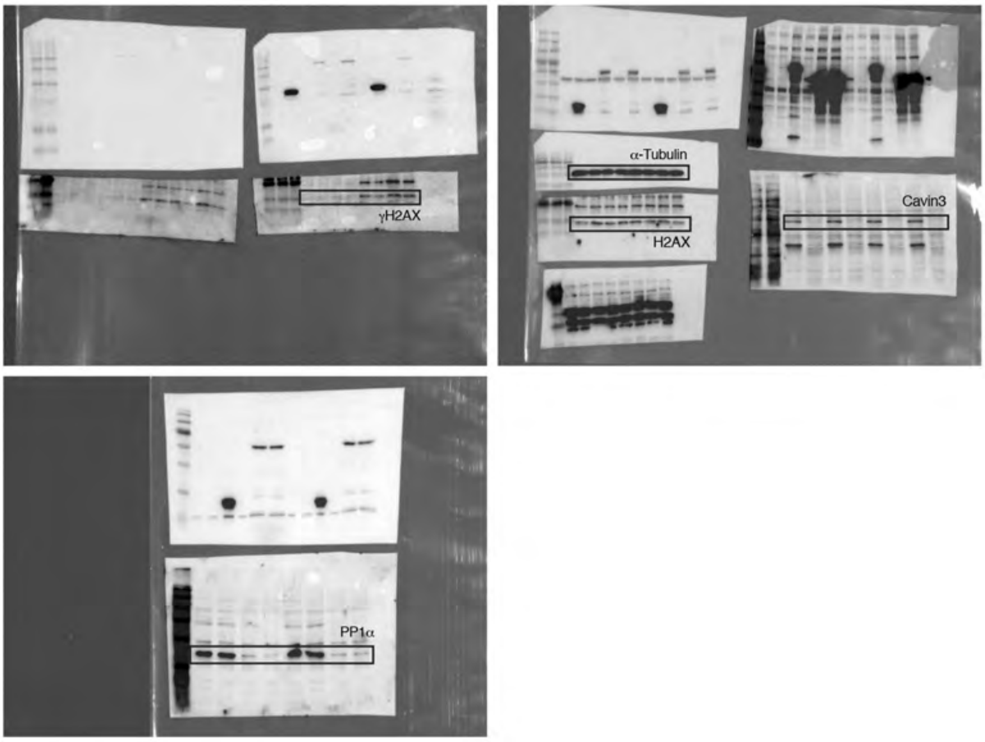

Figure 5f

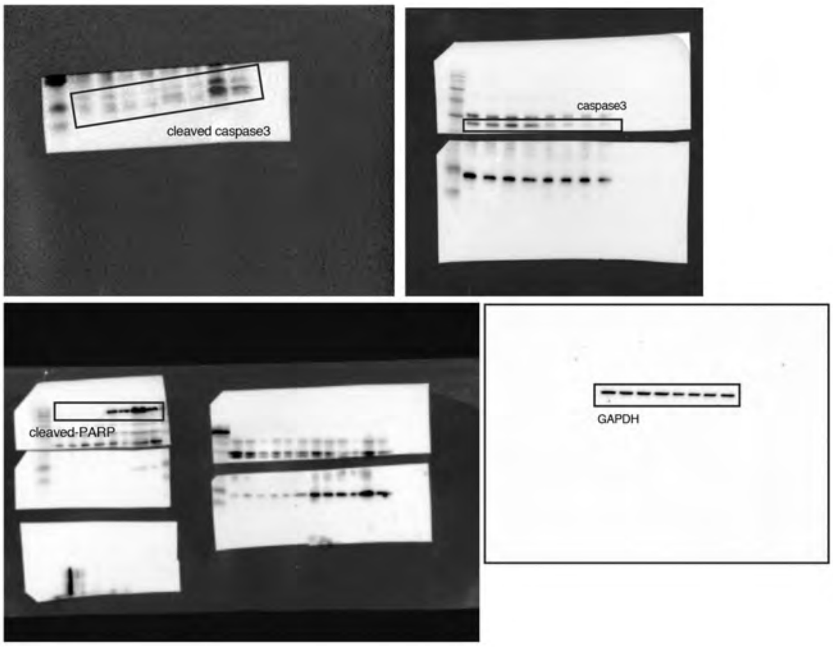

Supplementary Figure 11. Uncropped and unprocessed scans of the western blots.

Figure 6c

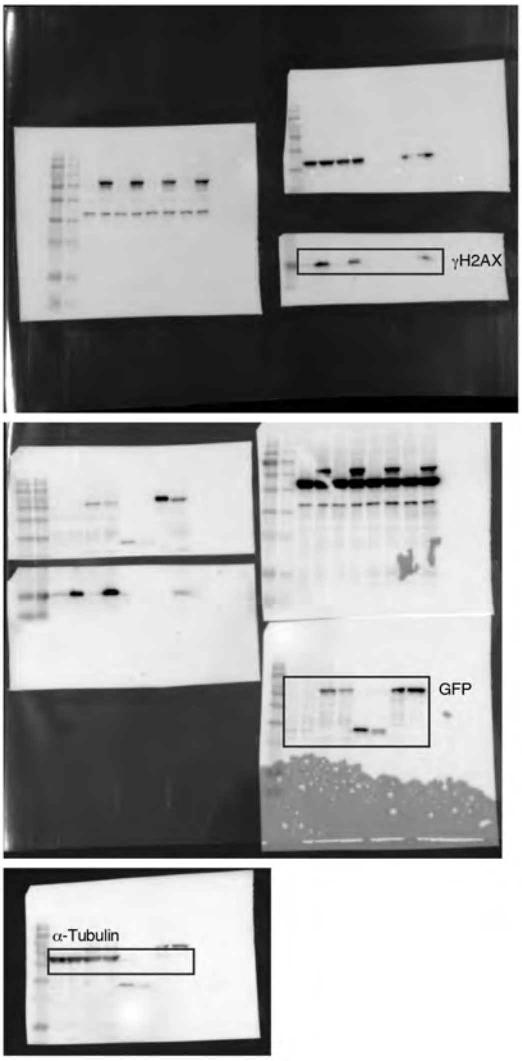

Figure 6d

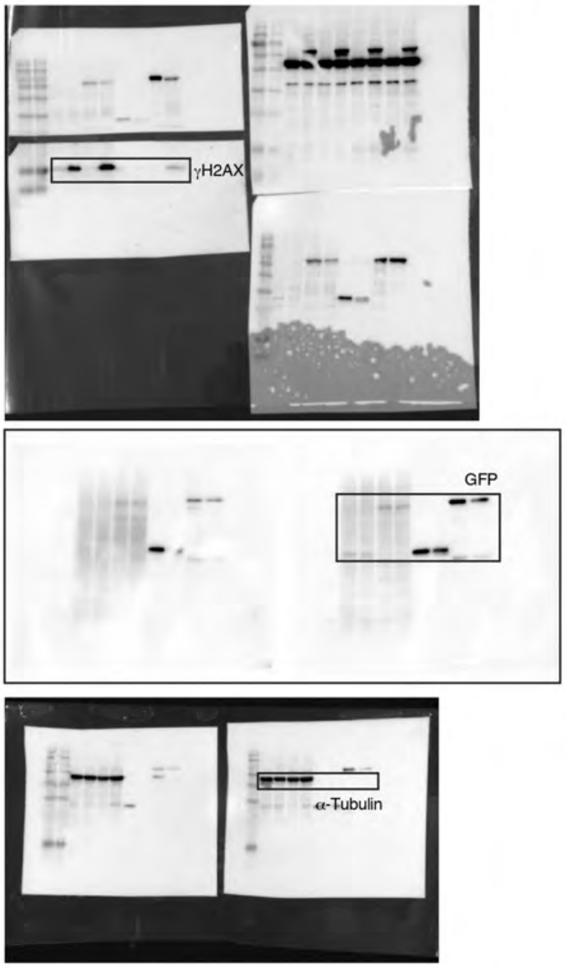

Figure 6a

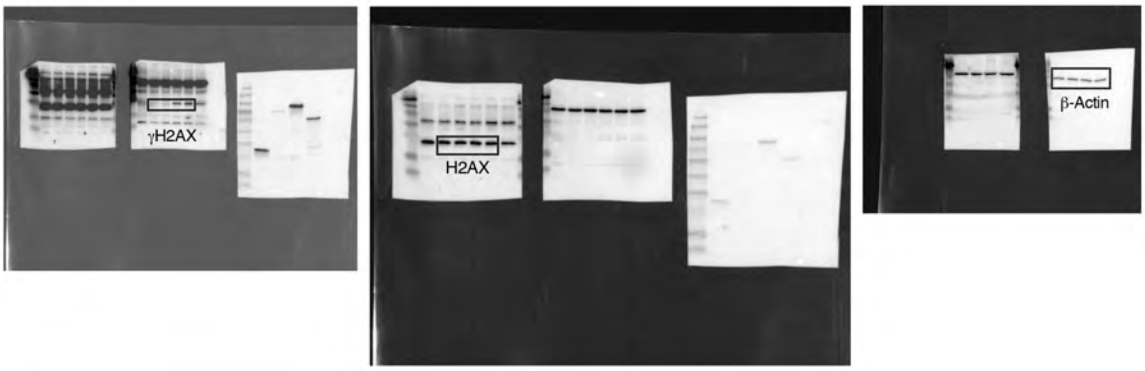

Supplementary Figure 11. Uncropped and unprocessed scans of the western blots.

Supplementary Figure 10b

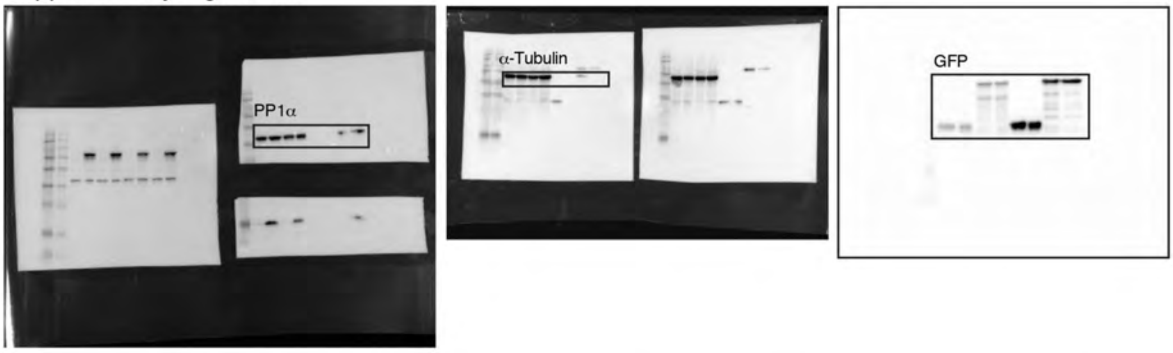

Supplementary Figure 10c

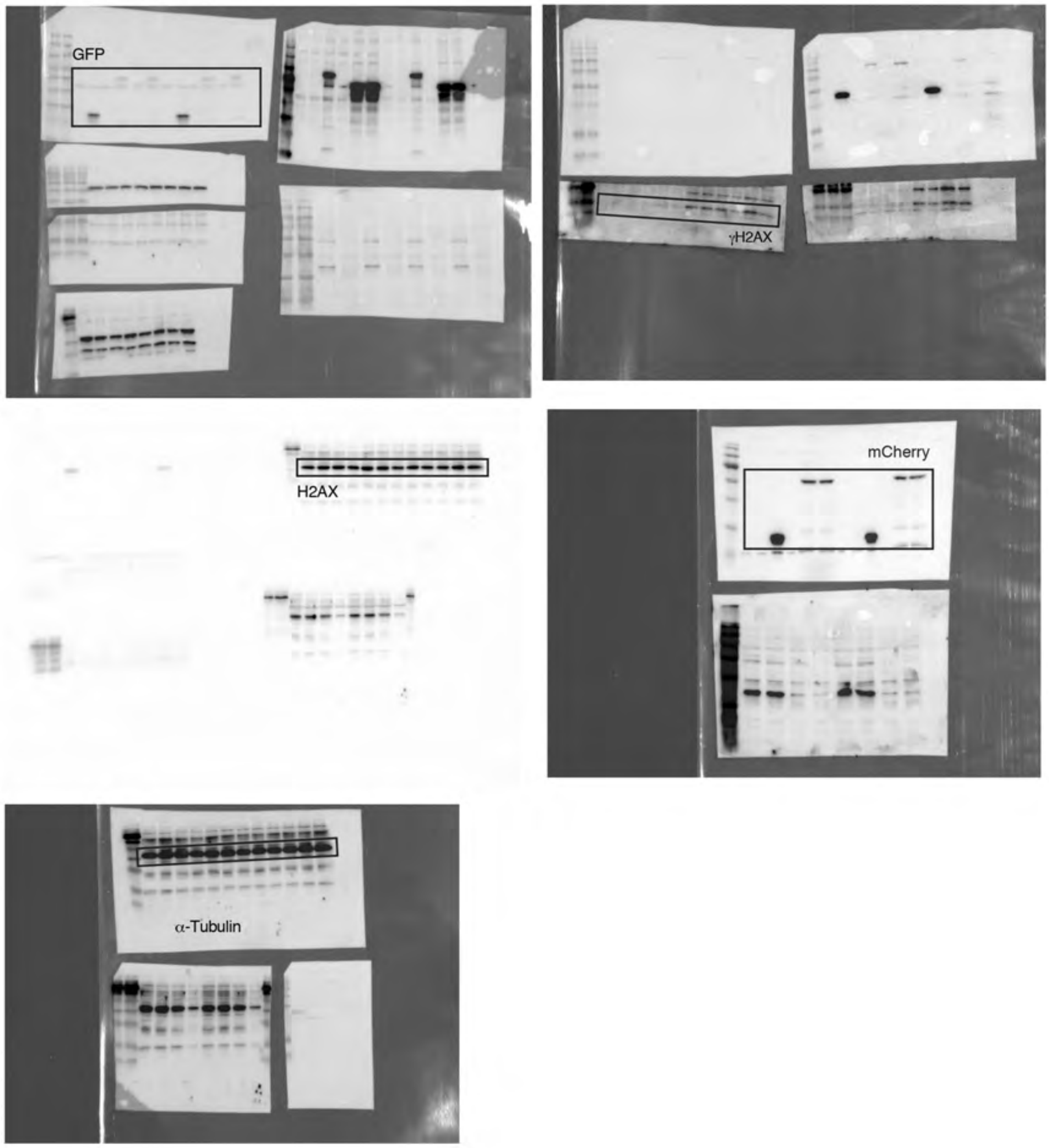

Supplementary Figure 11. Uncropped and unprocessed scans of the western blots.

Supplementary Figure 10e

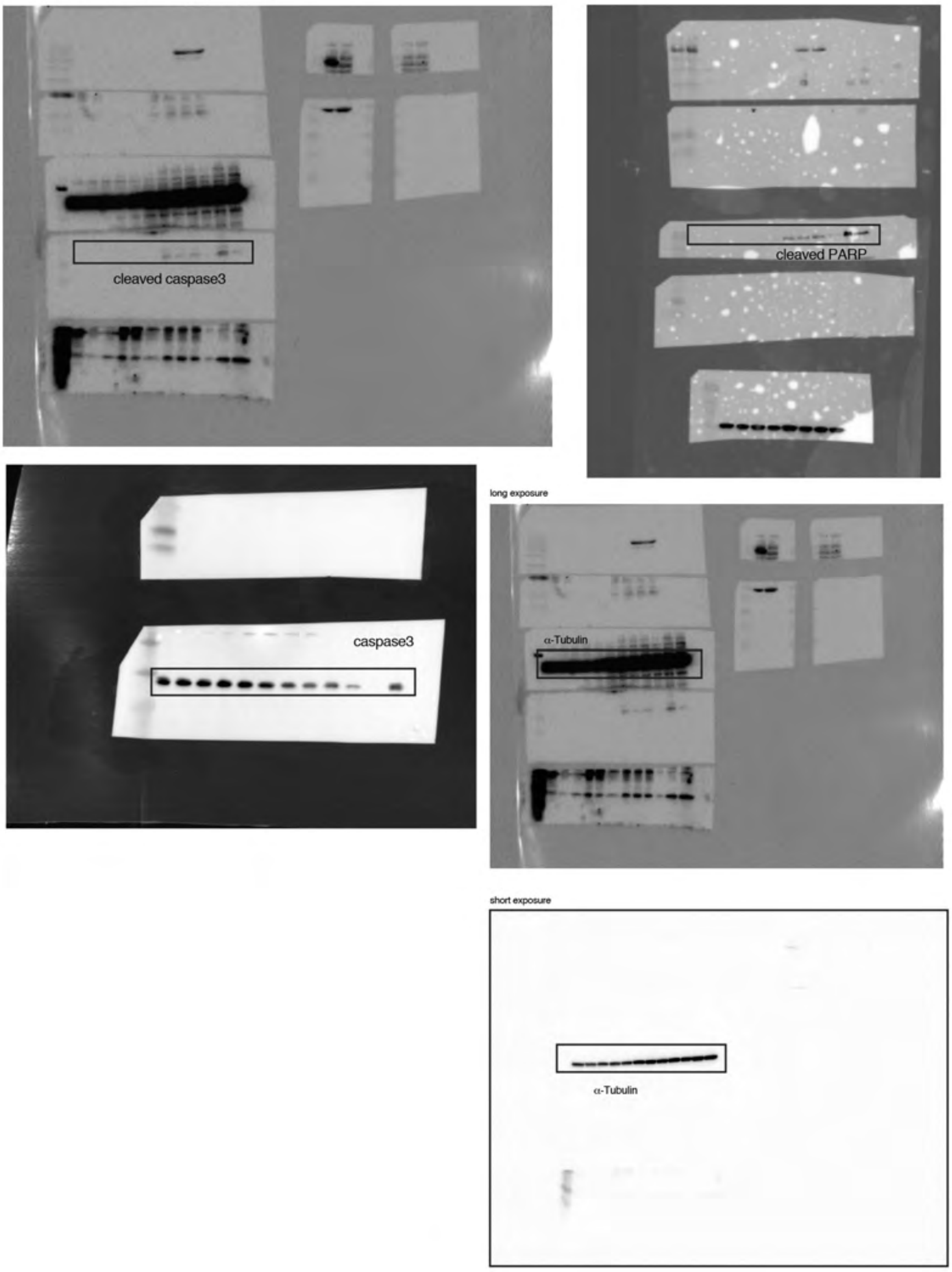

Supplementary Figure 11. Uncropped and unprocessed scans of the western blots.

Supplementary Table 1. Compiled list of published potential interacting proteins of Cavin1 and Cavin3.

	**Gene ID**	**Gene Description**	**Experimental evidence database**	**Supplementary References**
**CAVIN1**	BAG3	BCL2-associated athanogene 3	Affinity capture MS	1
	CRY2	Crytochrome 2	Affinity capture MS	2-3
	ELAV1	ELAV like RNA binding protein 1	Affinity capture RNA	4
	FLOT2	flotillin 2	Co-fractionation	5
	ILF2	Interleukin enhancer binding factor 2	Co-fractionation	5
	ILF3	Interleukin enhancer binding protein 3	Co-fractionation	5
	LIPE	Lipase, hormone-sensitive	Affinity capture-Western, Co-fractionation	6
	MYBBP1A	MYB binding protein 1a	Co-fractionation	5
	MYC	v-myc avian myelocytomatosis viral oncogene homolog	Affinity capture MS	7
	NHP2L1	NHP2 non-histone chromosome protein 2-like 1	Co-fractionation	5
	NOLC1	Nucleolar and coil-body phosphoprotein 1	Co-fractionation	5
	NOP56	NOP56 ribonucleoprotein	Co-fractionation	5
	PER2	Period2	Affinity capture MS	3
	RPS4X	Ribosomal protein S4, X-linked	Co-fractionation	5
	TMED10	Transmembrane emp24-like trafficking protein 10	Co-fractionation	5
	TRIM72	Tripartite motif containing 72, E3 ubiquitin protein ligase	Affinity capture-Western	8
**CAVIN3**	CRY2	Crytochrome 2	Affinity capture MS	3
	FLOT2	flotillin 2	Affinity capture MS	9
	ILF2	Interleukin enhancer binding factor 2	Co-fractionation	5
	ILF3	Interleukin enhancer binding protein 3	Co-fractionation	5
	MYC	v-myc avian myelocytomatosis viral oncogene homolog	Affinity capture MS	7
	PER2	Period2	Affinity capture MS	3

* Potential interacting proteins of Cavin1 and Cavin3 are listed as Gene ID, Gene Description, Experimental evidence database and published references that were used to form the basis of the protein list for the ALPHAScreen.

Supplementary Discussion: Potential functions for Cavin interacting proteins in cells

Potential non-caveolar cavin interacting proteins and their localization in the cell

The proteins identified as potential non-caveolar cavin interacting proteins are distributed throughout the cell, with 38% of identified proteins localized predominantly in the cytosol, 33% of proteins localized predominantly in the nucleus, 6% of proteins localized predominantly in the endoplasmic reticulum, plasma membrane and mitochondria, 3% of proteins localized predominantly to the cytoskeletal and nucleolus, 2% of proteins localized predominantly in the Golgi apparatus, and less than 1% of proteins localized predominantly to lysosomes, caveolae and peroxisomes, respectively. These data suggest that cavin proteins can interact with many other intracellular compartments when released from caveolae with a propensity for interaction with many nuclear and cytosolic proteins that freely shuttle between these two compartments in response to stress stimuli. For example, several proteomic studies have identified changes in the subcellular localization of Cavin1 and Cavin3 in response to cellular stressors including the oxidative stress induced nuclear accumulation of Cavin1 and Cavin3 in human fibroblasts (10,11). In addition, DDX21 and other DDX proteins (DDX1 and DDX5) as potential non-caveolar cavin proteins can translocate from the nucleolus to the nucleoplasm in response to stress stimuli (12). In the course of the review of this manuscript, a paper by Mendoza-Topaz et al. (13) applied BioID to identify proteins that interact with Cavin1. Candidate Cavin1-interacting proteins included a number of proteins located predominantly in the nucleus with functions related to the role of Cavin1 in regulating ribosomal RNA synthesis (14). Collectively, these findings suggest that the reach of the caveola function now extends from the plasma membrane to intracellular targets in compartments such as the cytoplasm, nucleus and nucleolus and specific cellular processes which could not have been envisaged before.

Metabolism and Protein Synthesis

The BioID/MS approach identified a number of key glycolytic enzymes as potential cavin-interacting proteins, including alpha enolase (ENO1), fructose bisphosphate aldolase A (ALDOA), pyruvate kinase (PKM) and phosphoglycerate kinase A (PGK1). Of particular interest is Pyruvate Kinase (PKM), the rate limiting enzyme in this process. These findings have potential importance for the role of cavins in cancer (15-19) as transformed cells predominantly metabolize glucose by glycolysis to produce energy in order to sustain their increased metabolic requirement, a process known as the Warburg effect (20). Recent reports suggest that the *in vitro* consequences of the loss of Cavin3 by gene knockout studies include the induction of Warburg metabolism (aerobic glycolysis) and the *in vivo* consequence of Cavin3 loss in a mouse model system is increased lactate production (21) suggesting that Cavin3 may play an important role in this process.

The metabolic shift from oxidative phosphorylation to aerobic glycolysis is partially achieved by a

switch in the splice isoforms of PKM from PKM1 to PKM2 that is demonstrated here as a potential Cavin3 interacting protein (22). Switching from PKM1 to PKM2 promotes aerobic glycolysis and thus provides a selective advantage for tumor formation. This switching mechanism that involves the generation of alternative splicing of two mutually exclusive exons for PKM is controlled by heterogenous nuclear ribonucleoprotein (hnRNP) family members along with the polypyrimidine tract binding protein (PTB; known also as hnRNPI) (23). Interestingly, BioID/MS analysis of potential Cavin3 interacting proteins identified heterogenous nuclear ribonucleoproteins (hnRNPs), suggesting intimate involvement of Cavin3 in determining the glycolytic phenotype of tumour cells. Furthermore, Cheng et al. (24) recently described a critical role for the eukarytic Elongation Factor-2 Kinase (eEF2-K) in the Warburg effect through controling synthesis of the protein phosphatase 2A (PP2A) A subunit. eEF2-K phosphorylates eukaryotic Elongation Factor 2 (eEF2) on Threonine (Thr)-56, thereby inactivating this key elongation factor that blocks protein translation and inhibits protein synthesis. Indeed, eEF2 was identified by the BioID/Mass Spectrometry analysis as a potential Cavin3 interacting protein. Additionally, eEF2-K confers tolerance to stress conditions in cancer cells (25). Collectively, these findings suggest involvement of Cavin3 in stress responsive pathways that influence a number of processes including metabolism and protein translation.

Supplementary Information References

1. Chen, Y. *et al.* Bcl2-associated athanogene 3 interactome analysis reveals a new role in modulating proteasome activity. *Mol Cell Proteomics* **12**, 2804-2819 (201

2. Hirano, A. *et al.* USP7 and TDP-43: Pleiotropic Regulation of Cryptochrome Protein Stability Paces the Oscillation of the Mammalian Circadian Clock. *PloS one* **11**, e0154263 (2016)

3. Schneider, K. *et al.* CAVIN-3 regulates circadian period length and PER:CRY protein abundance and interactions. *EMBO Rep* **13**, 1138-1144 (2012).

4. Abdelmohsen, K. *et al.* Ubiquitin-mediated proteolysis of HuR by heat shock. *The EMBO journal* **28**, 1271-1282 (2009).

5. Havugimana, P.C. *et al.* A census of human soluble protein complexes. *Cell* **150**, 1068-1081 (2012).

6. Aboulaich, N., Ortegren, U., Vener, A.V. & Stralfors, P. Association and insulin regulated translocation of hormone-sensitive lipase with PTRF. *Biochemical and biophysical research communications* **350**, 657-661 (2006).

7. Agrawal, P., Yu, K., Salomon, A.R. & Sedivy, J.M. Proteomic profiling of Myc-associated proteins. *Cell cycle* **9**, 4908-4921 (2010).

8. Zhu, H. *et al.* Polymerase transcriptase release factor (PTRF) anchors MG53 protein to cell injury site for initiation of membrane repair. *The Journal of biological chemistry* **286**, 12820-12824 (2011).

9. Hein, M.Y. *et al.* A human interactome in three quantitative dimensions organized by stoichiometries and abundances. *Cell* **163**, 712-723 (2015).

10. Baqader, N.O., Radulovic, M., Crawford, M., Stoeber, K. & Godovac-Zimmermann, J. Nuclear cytoplasmic trafficking of proteins is a major response of human fibroblasts to oxidative stress. *Journal of proteome research* **13**, 4398-4423 (2014).

11. Radulovic, M., Baqader, N.O., Stoeber, K. & Godovac-Zimmermann, J. Spatial Cross-Talk between Oxidative Stress and DNA Replication in Human Fibroblasts. *Journal of proteome research* **15**, 1907-1938 (2016).

12. Mialon, A. *et al.* Identification of nucleolar effects in JNK-deficient cells. *FEBS letters* **582**, 3145-3151 (2008).

13. Mendoza-Topaz, C., Yeow, I., Riento, K. & Nichols, B.J. BioID identifies proteins involved in the cell biology of caveolae. *PloS one* **13**, e0209856 (2018)

14. Liu, L. & Pilch, P.F. PTRF/Cavin-1 promotes efficient ribosomal RNA transcription in response to metabolic challenges. *Elife* **5** (2016).

15. Gupta, R., Toufaily, C. & Annabi, B. Caveolin and cavin family members: dual roles in cancer. *Biochimie* **107 Pt B**, 188-202 (2014).

16. Liu, L. *et al.* Cavin-1 is essential for the tumor-promoting effect of caveolin-1 and enhances its prognostic potency in pancreatic cancer. *Oncogene* **33**, 2728-2736 (2014).

17. Low, J.Y. & Nicholson, H.D. Emerging role of polymerase-1 and transcript release factor (PTRF/ Cavin-1) in health and disease. *Cell Tissue Res* **357**, 505-513 (2014).

18. Wang, Z. *et al.* Caveolin-1, a stress-related oncotarget, in drug resistance. *Oncotarget* **6**, 37135-37150 (2015).

19. Yi, J.S. *et al.* PTRF/cavin-1 is essential for multidrug resistance in cancer cells. *Journal of proteome research* **12**, 605-614 (2013).

20. Vander Heiden, M.G., Cantley, L.C. & Thompson, C.B. Understanding the Warburg effect: the metabolic requirements of cell proliferation. *Science* **324**, 1029-1033 (2009).

21. Hernandez, V.J. *et al.* Cavin-3 dictates the balance between ERK and Akt signaling. *Elife* **2**, e00905 (2013).

22. Zhan, C. *et al.* Isoform switch of pyruvate kinase M1 indeed occurs but not to pyruvate kinase M2 in human tumorigenesis. *PloS one* **10**, e0118663 (2015).

23. Chen, M., Zhang, J. & Manley, J.L. Turning on a fuel switch of cancer: hnRNP proteins regulate alternative splicing of pyruvate kinase mRNA. *Cancer research* **70**, 8977-8980 (2010).

24. Cheng, Y. *et al.* eEF-2 kinase is a critical regulator of Warburg effect through controlling PP2A-A synthesis. *Oncogene* **35**, 6293-6308 (2016).

25. Zhu, H. *et al.* Eukaryotic elongation factor 2 kinase confers tolerance to stress conditions in cancer cells. *Cell Stress Chaperones* **20**, 217-220 (2015).
