# Supplementary material for: Identification of intracellular cavin target proteins reveals cavin-PP1alpha interactions regulate apoptosis
Source: Nat Commun. 2019 Jul 22;10:3279. doi: 10.1038/s41467-019-11111-1 (PMC6646387; doi:10.1038/s41467-019-11111-1)
Supplement: Supplementary file 4 — Reporting Summary [file 41467_2019_11111_MOESM4_ESM.pdf]

nature research

Corresponding author(s): Robert Parton

Last updated by author(s): 2019-6-4

Reporting Summary

Nature Research wishes to improve the reproducibility of the work that we publish. This form provides structure for consistency and transparency in reporting. For further information on Nature Research policies, see Authors & Referees and the Editorial Policy Checklist.

Statistics

For all statistical analyses, confirm that the following items are present in the figure legend, table legend, main text, or Methods section.

n/a	Confirmed	
☐	☒	The exact sample size (*n*) for each experimental group/condition, given as a discrete number and unit of measurement
☐	☒	A statement on whether measurements were taken from distinct samples or whether the same sample was measured repeatedly
☐	☒	The statistical test(s) used AND whether they are one- or two-sided<br>*Only common tests should be described solely by name; describe more complex techniques in the Methods section.*
☒	☐	A description of all covariates tested
☒	☐	A description of any assumptions or corrections, such as tests of normality and adjustment for multiple comparisons
☐	☒	A full description of the statistical parameters including central tendency (e.g. means) or other basic estimates (e.g. regression coefficient) AND variation (e.g. standard deviation) or associated estimates of uncertainty (e.g. confidence intervals)
☐	☒	For null hypothesis testing, the test statistic (e.g. *F*, *t*, *r*) with confidence intervals, effect sizes, degrees of freedom and *P* value noted<br>*Give P values as exact values whenever suitable.*
☒	☐	For Bayesian analysis, information on the choice of priors and Markov chain Monte Carlo settings
☐	☒	For hierarchical and complex designs, identification of the appropriate level for tests and full reporting of outcomes
☐	☒	Estimates of effect sizes (e.g. Cohen's *d*, Pearson's *r*), indicating how they were calculated

Our web collection on statistics for biologists contains articles on many of the points above.

Software and code

Policy information about availability of computer code

Data collection	*Provide a description of all commercial, open source and custom code used to collect the data in this study, specifying the version used OR state that no software was used.*
Data analysis	*Provide a description of all commercial, open source and custom code used to analyse the data in this study, specifying the version used OR state that no software was used.*

For manuscripts utilizing custom algorithms or software that are central to the research but not yet described in published literature, software must be made available to editors/reviewers. We strongly encourage code deposition in a community repository (e.g. GitHub). See the Nature Research guidelines for submitting code & software for further information.

Data

Policy information about availability of data

All manuscripts must include a data availability statement. This statement should provide the following information, where applicable:

- Accession codes, unique identifiers, or web links for publicly available datasets
- A list of figures that have associated raw data
- A description of any restrictions on data availability

The source data underlying Figs. 1a, 2a-d, 6d, h and 7a and Supplementary Figures 1a and 5d are provided as a Source Data file. All reagents and further experimental data are available from the corresponding author upon reasonable request. Proteomics data that supports the findings of this study have been deposited in ProteomeXchange with the accession number Y.

Field-specific reporting

Please select the one below that is the best fit for your research. If you are not sure, read the appropriate sections before making your selection.

☒ Life sciences ☐ Behavioural & social sciences ☐ Ecological, evolutionary & environmental sciences

For a reference copy of the document with all sections, see nature.com/documents/nr-reporting-summary-flat.pdf

Life sciences study design

All studies must disclose on these points even when the disclosure is negative.

Sample size	For quantitative experiments, the number of cells and number of independent experiments analysed is stated in the Figure legends based on initial trials. The significance criterion i.e p<0.0001 in many experiment conditions and sample size i.e 150 cells analyzed per experiment for PLA experiments reflect adequate power to detect a pre-specified effect size.
Data exclusions	No data was excluded from the analysis.
Replication	All experiments were repeated three times unless stated in the Figure legends.
Randomization	No randomization was used to determine sample allocation. The control and treated groups of cells came from the same cell cultures and were treated and analysed at the same time.
Blinding	The investigators were blinded to allocation during experiments related to electron microscopy that is stated in the appropriate Methods section of the manuscript

Reporting for specific materials, systems and methods

We require information from authors about some types of materials, experimental systems and methods used in many studies. Here, indicate whether each material, system or method listed is relevant to your study. If you are not sure if a list item applies to your research, read the appropriate section before selecting a response.

Materials & experimental systems

n/a	Involved in the study
☐	☒ Antibodies
☐	☒ Eukaryotic cell lines
☒	☐ Palaeontology
☒	☐ Animals and other organisms
☒	☐ Human research participants
☒	☐ Clinical data

Methods

n/a	Involved in the study
☒	☐ ChIP-seq
☒	☐ Flow cytometry
☒	☐ MRI-based neuroimaging

Antibodies

Antibodies used

Anti Caveolin-1 antibody, BD Bioscience, Catalogue no: 610060, Clone no: polyclonal, Lot no: 3352931
Anti Cavin1 C1 antibody - made in house, rabbit polyclonal antibody to C terminus of Cavin1
Anti Cavin3 antibody, ProteinTech Group, Catalogue no: 16250-1-AP, clone number: rabbit polyclonal IgG, immunogen catalogue no: Ag9209
Anti Cavin1 antibody, SigmaAldrich, Catalogue no: AV36965, clone name: rabbit polyclonal, lot no: QC6676
Anti Cavin3 antibody, Novus Biologicals, Catalogue no: HOO112464-MO4, clone name: (161-261) mAb, lot no: 11266-8D3
Anti PP1 alpha antibody FL-18, Santa Cruz Biotechnologies, Catalogue no: sc-443, clone no: FL-18, rabbit IgG, lot no: D1713
Anti PP1 alpha antibody G-4, Santa Cruz Biotechnologies, Catalogue no: sc-271762, clone name: G-4 mouse monoclonal , lot no: H1213
Anti Cleaved Caspase 3 (Asp175) antibody, Cell Signaling Technology, Catalogue no: 9661, clone name: rabbit polyclonal
Anti Caspase 3 antibody, Cell Signaling Technology, Catalogue no: 8G10, clone name: rabbit mAb, lot no: 3
Apoptosis and DNA damage H2AX (S139) and cleaved PARP and anti-GAPDH Western blot cocktail antibody, Abcam Australia, Catalogue no: ab131385, clone name: mouse monoclonal, lot no: J5378
Anti yH2AX (P139) antibody, Abcam Australia, Catalogue no: ab2893, clone name: rabbit polyclonal, lot no: GR3242597-1
Anti H2AX total antibody, Abcam Australia, Catalogue no: clone name: rabbit polyclonal, lot no: GR269626-11
Anti GFP antibody, Roche, Catalogue no: 1181446001, Clone name: Clone 7.1 and 13.1, lot no: 14442000
Anti Tubulin antibody, Sigma Aldrich, Catalogue no: T9026, clone DM1A
Anti-Cherry antibody, BioVision, Catalogue no: 5411, clone no: clone 1, lot no: 2M02L54110
Anti-GAPDH antibody, Invitrogen by ThermoFisher Scientific, clone name: 6C5, Catalogue no: AM4300, lot no: 00439919

Validation

Anti-GAPDH antibody, Invitrogen, has been referenced in more than 250 publications for Western blot analysis as a loading

control
Anti-Caveolin-1 BD Bioscience antibody, Catalogue no: 610060, has been routinely used in the laboratory for more than 10 years and is considered the gold standard for Caveolin-1 specific antibodies.
Anti-Cavin1 C1 antibody was made in house and has been used in several peer review publications from our laboratory
Anti-Cavin3 ProteinTech antibody has been used in 15 peer review publications and has been validated in several Cavin3 KO cell lines
Cavin3 and PP1 antibodies were validated in the paper using siRNA mediated knockdown of the respective proteins followed by Western analysis with the antibodies listed above.
Anti Cleaved Caspase 3 (Asp175) antibody from Cell Signaling Technology has over 3500 citations according to the manufacturers website
Anti Caspase 3 antibody from Cell Signaling Technology has over 1500 citations according to the manufacturers website
Anti yH2AX (P139) Abcam antibody has been referenced in 176 peer review publications
Anti yH2AX Abcam antibody has been referenced in 95 peer review publications
Anti GFP antibody from Roche has been referenced in over 300 peer review publications.
Anti-Tubulin antibody from Sigma Aldrich, Catalogue no: T9026 has been used in over 1000 peer review publications

Eukaryotic cell lines

Policy information about cell lines

Cell line source(s)	All cell lines were purchased from ATCC: MCF-7 cells (ATCC HTB-22), A431 cells (ATCC CRL-1555) and MDA MB 231 cells (ATCC HTB-26)
Authentication	MCF-7 cells were subjected to STR profiling at QIMR Berhofer Medical Research Institute
Mycoplasma contamination	All cell lines were subjected to routine mycoplasma testing every three months and were negative for mycoplasma contamination.
Commonly misidentified lines (See ICLAC register)	No commonly misidentified cell lines were used in this study
